# A Genomic-Based Approach Combining *In Vivo* Selection in Mice to Identify a Novel Virulence Gene in *Leishmania*

**DOI:** 10.1371/journal.pntd.0000248

**Published:** 2008-06-11

**Authors:** Wen-Wei Zhang, Christopher S. Peacock, Greg Matlashewski

**Affiliations:** 1 Department of Microbiology and Immunology, McGill University, Montreal, Quebec, Canada; 2 Wellcome Trust Sanger Institute, Wellcome Trust Genome Campus, Hinxton, Cambridgeshire, United Kingdom; Yale University, United States of America

## Abstract

**Background:**

Infection with *Leishmania* results in a broad spectrum of pathologies where *L. infantum* and *L. donovani* cause fatal visceral leishmaniasis and *L. major* causes destructive cutaneous lesions. The identification and characterization of *Leishmania* virulence genes may define the genetic basis for these different pathologies.

**Methods and Findings:**

Comparison of the recently completed *L. major* and *L. infantum* genomes revealed a relatively small number of genes that are absent or present as pseudogenes in *L. major* and potentially encode proteins in *L. infantum*. To investigate the potential role of genetic differences between species in visceral infection, seven genes initially classified as absent in *L. major* but present in *L. infantum* were cloned from the closely related *L. donovani* genome and introduced into *L. major*. The transgenic *L. major* expressing the *L. donovani* genes were then introduced into BALB/c mice to select for parasites with increased virulence in the spleen to determine whether any of the *L. donovani* genes increased visceral infection levels. During the course of these experiments, one of the selected genes (LinJ32_V3.1040 (Li1040)) was reclassified as also present in the *L. major* genome. Interestingly, only the Li1040 gene significantly increased visceral infection in the *L. major* transfectants. The Li1040 gene encodes a protein containing a putative component of an endosomal protein sorting complex involved with protein transport.

**Conclusions:**

These observations demonstrate that the levels of expression and sequence variations in genes ubiquitously shared between *Leishmania* species have the potential to significantly influence virulence and tissue tropism.

## Author Summary

Parasites of the genus *Leishmania* cause a variety of human diseases that range from destructive skin lesions caused by *L. major* to visceral infections of the liver and spleen caused by *L. donovani* that result in death. The *Leishmania* genes responsible for these different pathologies are not known. In the present study, we used a comparative genome-based approach to introduce and over-express *L. donovani* genes in *L. major* to determine whether this results in increased virulence of *L. major* in visceral organs of infected mice. Through this approach, a novel gene termed Li1040 was identified that is potentially involved in protein transport and was shown to increase pathogenesis in the visceral organs in mice. The Li1040 gene may therefore represent a *Leishmania* virulence gene that has the potential to regulate the pathology of infection in the mammalian host. These observations help to define how *Leishmania* causes fatal infections in humans and therefore provide a parasite-specific target for therapy.

## Introduction

*Leishmania* protozoa are transmitted by the bite of an infected sandfly and cause a spectrum of diseases ranging from self-healing cutaneous lesions to fatal visceral infection [1],[2]. There are an estimated 12 million cases in over 80 countries, with an annual incidence of 0.5 million new cases of the visceral leishmaniasis and 2.0 million cases of the cutaneous leishmaniasis [3]. More than 20 different *Leishmania* species can infect humans. Host health status and genetic background can influence the outcome of infection [4],[5] and HIV co-infection has dramatically increased the incidence of visceral leishmaniasis [5]. The major factor that determines the tropism and pathology of *Leishmania* infection is however the species of *Leishmania* [1],[2]. For example, *L. donovani*, *L. infantum* and *L. chagasi* are closely related members of the *L. donovani* complex that cause visceral leishmaniasis, which is fatal if not treated. *L. major* and *L. tropica* infections usually result in cutaneous lesions that remain localized at the site of the sandfly bite. *L. (Viannia) braziliensis* causes cutaneous leishmaniasis but can also migrate from the site of initial infection to the nasopharyngeal area resulting in highly destructive mucocutaneous leishmaniasis. The *Leishmania* genome projects are expected to help identify the genetic differences between these parasites which govern the pathology and tropism of infection caused by the different *Leishmania* species [6]–[8].

Our laboratory has previously identified the A2 gene family, which is present in *Leishmania* species that cause visceral infections including *L. infantum* and *L. donovani* but are not present in many of the *Leishmania* species that cause cutaneous infections including *L. major* and *L. tropica* [9],[10]. A2 proteins have been shown to be essential for visceral infection with *L. donovani* in BALB/c mice [11],[12]. Cross species transfection of the A2 gene from *L. donovani* into *L. major* rendered *L. major* more virulent in visceral organs but less virulent at cutaneous sites, phenotypes typical of *L. donovani* [12],[13]. This demonstrated that species-specific genes can play a role in virulence and the pathology of *Leishmania* infection and provided the justification for experimentally studying species-specific genes identified through sequencing of the *Leishmania* genomes.

We used the genetic information from the completion of the *L. major*, *L. infantum*, and *L. braziliensis* genomes [6],[7] to identify genes that could potentially influence the pathology caused by these *Leishmania* species. Remarkably, out of more than 8000 genes within the *Leishmania* genome, only about 25 *L. infantum*-specific genes have been identified which are not present or are pseudogenes in *L. major* and *L. braziliensis*, and most encode for proteins with no known function [7].

Using the *L. infantum* genome sequence database, we have cloned 7 *L. donovani* ortholog genes that were absent or were pseudogenes in *L. major* and introduced these genes into *L. major*. The *L. donovani* gene containing transgenic *L. major* parasites were introduced into BALB/c mice to determine whether any of these genes increased virulence in visceral sites including the liver and spleen. During the course of this study, one of these selected *L. infantum* genes, LinJ32_V3.1040 (Li1040), initially classified as absent in *L. major*, was identified in a blast search of the *L. major* shotgun sequences and subsequently reclassified as present in the genomes of *L. major*, *L. infantum*, *L. braziliensis* and *L. donovani*. Surprisingly, it was the Li1040 gene, which encodes a hypothetical protein potentially involved in protein transport, which dramatically increased *L. major* infection levels in the liver and spleen of BALB/c mice. These observations establish the functional genomic approach to study virulence genes in *Leishmania* and demonstrate that the levels of expression and/or sequence variation in genes conserved among different *Leishmania* species have the potential to contribute significantly to virulence and tissue tropism.

## Materials and Methods

### Comparison of *Leishmania infantum* and *Leishmania major* genomes

The detail comparison of three sequenced *Leishmania* genomes have been described [7]. However, gene-by-gene comparisons for this study were made in the first release of the *L. infantum* genome sequence (September 17 2004), following automatic annotation of translational open reading frames via BLAST analysis and comparison with *L. major*. This first stage analysis has demonstrated high conservation in both gene content and order (synteny) between the *L. infantum* and *L. major* genomes. At start of this study, only up to 20 genes have been identified that are either present in *L. infantum* and absent in *L. major* or which are present as complete open reading frames in *L. infantum* but occur as pseudogenes in *L. major*. In this study, we chose seven of these potential *L. infantum* specific genes, which we cloned from *L. donovani* 1S/Cl2D using primer sequences derived from the corresponding *L. infantum* genes. This *L. donovani* strain causes visceral infection in mice when introduced intravenously. The cloned *L. donovani* genes were subsequently transfected into *L. major* for crossing species transfection studies (Table 1) although one of these, Li1040 was later identified to be also present in *L. major* and *L. braziliensis*.

**Table 1:** *Leishmania* Genes Used in This Study.

*L.infantum*	*L.major*	*L.braziliensis*	Predicted identity	MW kDa
LinJ08_V3.0560	none	LbrM08_V2.0590	Cyclopropane fatty acyl phospholipid synthase	55.5
LinJ36_V3.0640	none	LbrM35_V2.0690 (pseudogene)	Sec14 cytosolic factor (phosphatidylinositol/phosphatidylc holine transfer protein)	48.5
LinJ15_V3.1370	none	LbrM15_V2.1300	Hypothetical protein	40.7
LinJ32_V3.1040 (Li1040)	LmjF32.0985 (Lm0985)	LbrM32_V2.1080	Hypothetical protein with Vps23_ core domain of Tsg101 protein	45.1
LinJ32_V3.1580	none	LbrM32_V2.1680 (pseudogene)	Hypothetical protein	28.5
LinJ36_V3.4190	none	LbrM35_V2.4235 (pseudogene)	Hypothetical protein	78.4
LinJ08_V3.0140	LmjF08.0135 (pseudogene)	LbrM08_V2.0140 (pseudogene)	Hypothetical protein with calcium-binding EF-hand domain	63.4

### Construction of *Leishmania* expression vectors containing A2 tag (pLA2tag) or GFP tag (pLGFPC)

To detect *L. donovani* gene products expressed in *L. major*, we modified the *Leishmania* expression vector (pLPneo) [14] by adding a 10 amino acid A2 epitope tag or a GFP tag into its multiple cloning site. Briefly, the pLA2tag vector was constructed by inserting an adapter sequence encoding 10 A2 amino acids (QSVGPLSVGP) and a stop codon flanked by *BamH* I and *Not* I sites into the multiple cloning sites (*BamH* I and *Not* I) of the pLPneo vector. The adapter sequences are: 5′ GATCCGCAGTCCGTCGG CCCGCTCTCCGTTGGCCC GTAGC (plus strain) and 5′GGCCGCTACGGGCCAAC GGAGAGCGGGCCGACGGACTGCG (minus strain). pLA2tag vector is therefore suitable for expressing fusion protein with A2 tag at its C-terminus. pLGFPC vector was constructed by following two steps: 1) a 820 bp *Nhe* I and *Bcl* I fragment containing green fluorescent protein (GFP) gene was removed from pEGFP-C3 vector (BD Biosciences Clontech). 2) the 820 bp *Nhe* I and *Bcl* I fragment was ligated into the *Xba* I and *BamH* I sites of pLPneo vector, generating pLGFPC vector. pLGFPC is suitable for expressing GFP fusion proteins with GFP at the N terminus.

### PCR amplification of *L. donovani* genes using primers based on the *L. infantum* gene sequences

To facilitate cloning of *L. donovani* genes into pLA2tag or pLGFPC vector, a restriction enzyme site (*Hind* III, *BamH* I or *Bgl* II) was added to the 5′ end of PCR primers. The PCR primers for LinJ32_V3.1040 are 5′cccaagcttACAATGGAGCTGACACTGCATC, 5′cgagatctGTGGGGAACATCATCTTGAGCTG and 5′cgagatctTAGGGGAACATC ATCTTGAGCTG; primers for LinJ08_V3.0560 are 5′cccaagcttCCAAGCTTCCAAGCATGGAAAACCGGCCA and 5′cgggatccgtCGGCCGGTACACGCTGACGTA; primers for LinJ36_V3.0640 are 5′cccaagcttCTCACCATGGCGGCAACTCATC and 5′cgggatcccaCTT CGGCAAACCGTTCTTTCG; primers for LinJ32_V3.1580 are 5′cccaagcttTCAAGCATGAGCACCAGTGCAG and 5′cgagatctGTCTTGTGACGCAATGGACCGATG; Primers for LinJ15_V3.1370 are 5′cccaagcttGGTAAGACGACTATGCGCAGCAG and 5′cgagatctGTACCGGCGAAGTAGCTGTGCAG; Primers for LinJ36_V3.4190 are 5′cccaagcttGCGATGGGGCGAATCGACTC and 5′cgagatctgtAGAGTTAG TCGGCAGCCGAGG: Primers for LinJ08_V3.0140 are 5′cccaagcttAACATTATG TTGGCTAGCGCTG and 5′cgagatctgtGAGCAGATTCGCAGCACGCA; Primers for LmjF32.0985 are 5′cccaagcttACAATGGAGCTGACACTGCATC and 5′cgagatctGT TTGGGTGAACATCATCTTGAGCTG. PCR amplifications were performed using Taq DNA polymerase (Invitrogen) following manufacturer's instruction.

### Parasite strains and culture conditions

*L. major* Friedlin V9, *L. donovani* 1S/Cl2D strains were used in this study. Promastigotes were routinely cultured at 27°C in M199 medium (pH 7.4) supplemented with 10% heat-inactivated fetal bovine serum, 40 mM Hepes (pH 7.4), 0.1 mM Adenine, 5 mg l^−1^ Haemin, 1 mg l^−1^ Biotin, 1 mg l^−1^ Biopterine, 50 U ml^−1^ Penicillin and 50 µg ml^−1^ Streptomycin. To determine the Li1040 gene transcript levels in different culture conditions, *L. donovani* and *L. major* promastigotes were also shifted to 37°C, pH 5.5 culture media for 6 hours to mimic the macrophage phagolysosome environment associated with the amastigote stage. Under these conditions, *L. donovani* remains viable and is induced to differentiate into amastigotes. Although the majority of *L. major* remain viable for this 6 hours period, they are unable to differentiate into amastigotes [13].

### Transfection procedure

The procedure for transfection was as previously described [14]. Briefly, 10–20 µg of plasmid DNA was used in each transfection. After electroporation, the *Leishmania* promastigotes were transferred into a drug-free culture medium and the following day, G418 was added to make the final concentration of G418 100 µg ml^−1^. To avoid selection of spontaneous mutants, pooled transfectants were used for all subsequent studies including mice infections, growth in culture, Southern , Northern and Western blot analysis.

### In vivo infection and selection for survival in BALB/c mice spleen

Female BALB/c mice weighing 18–20 g were purchased from Charles River Breeding Laboratories and maintained in the animal care facility under pathogen-free conditions. BALB/c mice were infected by tail vein injection with 1×10^8^ stationary-phase promastigotes in 100 µl PBS per mouse [11]–[13]. Six weeks post infection, the *in vivo* infection-selected amastigotes were isolated from the spleen as described [15]. The isolated amastigotes were transformed into promastigotes in M199 *L. major* culture medium containing 50 µg ml^−1^ G418. When the G418-resistant culture was established, the culture was subjected to Westernblot analysis. To subsequently compare the virulence of plasmid transfectants, BALB/c mice were infected by tail vein injection with 1×10^8^ stationary-phase promastigotes in 100 µl of PBS per mouse. The amastigotes were isolated from infected mice after 4, 6, 8 and 10 weeks of visceral infection respectively. The recovered amastigotes were cultured in promastigote culture medium, and the *Leishmania* parasite burdens were determined by limiting dilution. For cutaneous infections, mice were infected subcutaneously with 5×10^6^ stationary-phase promastigotes in their hind footpads. Disease progression was monitored by weekly caliper measurement of footpad swelling.

### Measurement of in vitro growth rates

The growth curves of *Leishmania* transfectants were measured in 96-well plates. Promastigotes in stationary phase were seeded at a concentration of 4×10^5^ ml^−1^ into wells containing 200 µl of medium with 50 µg ml^−1^ G418. Each sample was plated in triplicate. The OD_600_ values were measured daily for total 7 days.

### Western, Southern and Northern blot analysis

Total *Leishmania* protein, RNA, genomic DNA were prepared and analysed as previously described [16]. The DNA probes were labelled with [α-32P]-dCTP by random priming.

### Immunofluoresence microscopy

Indirect immunofluoresence was performed as described [14],[17],[18]. Anti-A2 monoclonal antibody C9 hybridoma supernatant without further dilution was used as the primary antibody in the immunofluoresence study.

## Results

### Selection for *Leishmania* genes that increase survival in visceral organs in BALB/c mice

**Figure 1:**
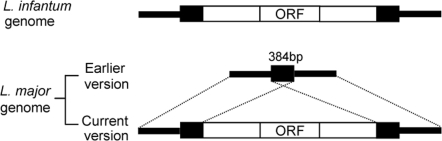
Comparison of Li1040 loci in *L. infantum* and *L. major*. Blast searching *L. major* shotgun sequences and Southern blot analysis indicated that the Li1040 ortholog gene is present in *L. major*. The earlier initial version of the *L. major* genome sequences contained only one of the flanking 384 bp repeat sequences, which flanks the Li1040 ortholog gene in *L. infantum* and *L. major*. Open reading frame: ORF.

At the beginning of this study, about 20 genes were identified as present in *L. infantum* and absent or were pseudogenes in the *L. major* genome. Among these genes, only a few encode products whose function could be predicted by sequence similarity searches. To investigate whether these species-specific genes are involved in tropism and pathology of *Leishmania* infection, 7 genes were initially selected for expression in *L. major*. This selection included 4 genes intact only in *L. infantum*, 2 genes intact in *L. infantum* and *L. braziliensis* but absent in *L. major*, and 1 gene which was intact in *L. infantum*, *L, braziliensis*, and *L. major* (Table 1). The single gene (Li1040) that is intact in all species was initially classified as absent in *L. major* but during the course of this study was reclassified as present in all *Leishmania* species. It is likely that the Li1040 gene was initially classified as absent in *L. major* during the assembly of the genome because it is flanked by two identical 384 repeat sequences (Figure 1).

**Figure 2:**
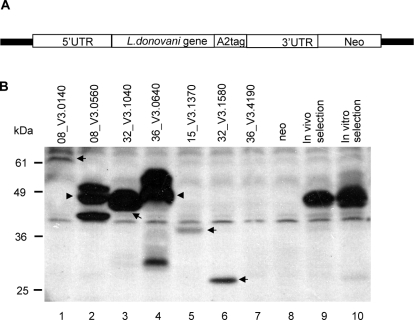
*L. major* transfectants containing *L. donovani* Li1040 ortholog were selected after *in vivo* infection and *in vitro* culture selections. A. Diagram of the pLA2tag vector used to express and detect *L. donovani* gene products in *L. major*. B. Expression of *L. donovani* genes in *L. major* and *in vivo* and *in vitro* selection of *L. major* transfectants. Individual *L. donovani* genes were cloned into the expression vector pLA2tag, expressed in *L. major* and detected by Western blot analysis with anti-A2 epitope tag antibodies (Lanes 1–8). For *in vivo* selection, the *L. major* transfectants shown in lanes 1–8 were pooled in equal numbers, injected into the tail vein of BALB/c mice, and 6 weeks later were isolated from the spleen and subjected to Western blot analysis with anti-A2 monoclonal antibodies (Lane 9). For *in vitro* selection, the *L. major* transfectants shown in lanes 1–8 were pooled and placed in amastigote growth conditions (pH5.5 and 37°C) for 3 days and then subjected to Western blot analysis with anti-A2 monoclonal antibodies (lane 10). Note: 6 out of these 7 *L. donovani* genes were expressed in *L. major* at the expected size indicated by arrows. The *L. major* transfectants expressing the *L. donovani* Li1040 ortholog became dominant after both *in vivo* and *in vitro* selections (Lanes 9 &10).

*L. infantum* and *L. donovani* both belong to the *L. donovani* species complex. We thus assumed that genes present in *L. infantum* would be largely identical in *L. donovani* and therefore the corresponding *L. donovani* genes were introduced into *L. major*. We carried out PCR amplification of the *L. infantum* ortholog genes from *L. donovani* 1S/Cl2D genomic DNA and ligated them into a *Leishmania* expression vector, pLPneo [14], that was engineered to encode a 10 amino acid *L. donovani* A2 peptide epitope tag at the C terminus as detailed in Methods (Figure 2A). Since *L. donovani* A2 proteins are absent in *L. major* [8],[10],[11], inclusion of the 10 amino acid A2 peptide epitope tag enabled detection of the *L. donovani* transgene products expressed in *L. major* using an anti-A2 monoclonal antibody (Mab). Expression of these *L. donovani* genes in transfected *L. major* were determined by Western blot analysis with anti-A2 Mabs and revealed that 6 out of the 7 selected genes expressed the corresponding proteins at the predicted molecular weights (Figure 2B, lanes 1–8). All of the transgenic *L. major* parasites except the LinJ36_V3.4190 transfectant stably expressed detectable levels of the *L. donovani* ortholog proteins.

We initially performed an *in vivo* selection in BALB/c mice to determine whether any of the transgenic *L. major* parasites were better adapted for survival in visceral organs. The transgenic *L. major* parasites including the vector control (Neo) were pooled, and injected into the tail vein of BALB/c mice. Amastigotes were recovered from the spleens of infected mice 6 weeks following injection, cultured out as promastigotes and subjected to Western blot analysis with anti-A2 epitope tag Mab to determine whether any of the *L. donovani* transgenes were expressed in the spleen derived parasites. As shown in Figure 2B, lane 9, the transgenic *L. major* parasites expressing the *L. donovani* Li1040 ortholog gene were detectable. This suggests that, relative to the other transfectants, the transgenic *L. major* expressing the *L. donovani* Li1040 ortholog gene displayed enhanced survival in the spleen.

In addition to the *in vivo* selection in BALB/c mice, we also performed an *in vitro* selection in axenic amastigote culture media. The pooled transgenic *L. major* promastigotes were placed in amastigote culture conditions at pH 5.5 and 37°C for 3 days, conditions that mimic the phagolysosomal compartment of macrophage cells in visceral organs. These culture conditions typically result in loss of viability of *L. major*, which are unable to adapt and proliferate under these conditions. In contrast, *L. donovani* differentiate into amastigote like parasites and are able to proliferate under these culture conditions. Following this *in vitro* selection, some of the transgenic *L. major* survived and were shifted back to promastigotes culture conditions (pH 7.4, 27°C) to allow them to proliferate. The resulting *in vitro* selected transgenic *L. major* was subjected to Western blot analysis with the anti-A2 epitope Mabs to detect expression of the *L. donovani* transgene products. As shown in Figure 2B lane 10, similar to the *in vivo* infection selection, the Li1040 protein was the major transgene product detectable in the *in vitro* selected transgenic *L. major*. Taken together, the *in vivo* and *in vitro* selection protocols resulted in selection for *L. major* parasites expressing the Li1040 ortholog gene.

### The Li1040 ortholog gene increases parasite numbers in visceral organs

**Figure 3:**
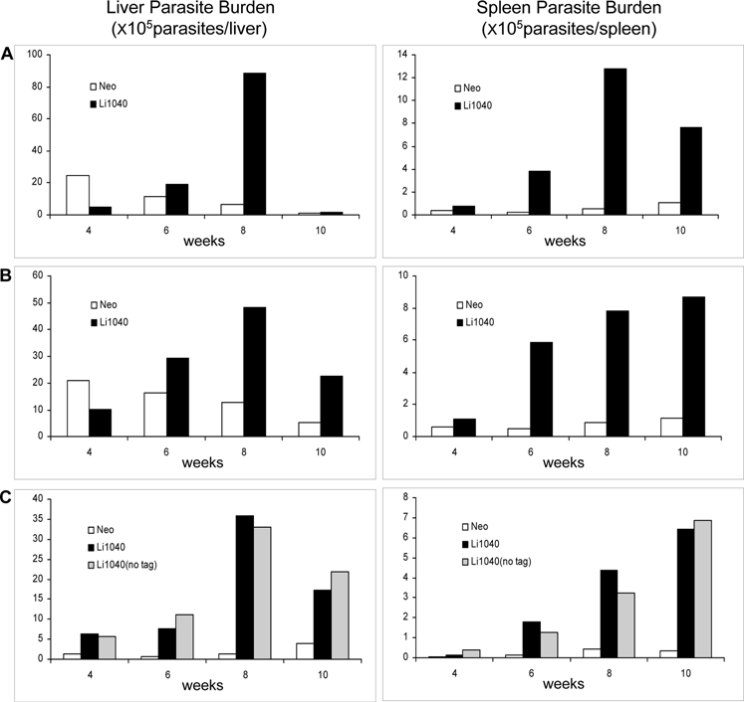
The *L. major* transfectants expressing the *L. donovani* Li1040 ortholog gene (Li1040) displayed increased virulence in visceral infection in mice. *L. major* expressing the *L. donovani* Li1040 ortholog with tag or no tag and *L. major* containing the control pLA2tag vector (Neo) were used to infect BALB/c mice by tail vein injection. The liver and spleen parasite burden levels were determined after 4, 6, 8 and 10 weeks after infection. Replica experiments are shown in Panels A and B. The effect of Li1040 protein with no tag on *L. major* visceral infection was also examined and compared with Li1040 protein with the A2 tag (Panel C).

The preceding experiments argue that expression of the *L. donovani* Li1040 gene ortholog in *L. major* provided a survival advantage in the spleen of BALB/c mice. It was therefore necessary to directly confirm this by comparing parasite numbers following infection with *L. major* expressing the Li1040 ortholog to the control *L. major* transfectants (Neo) containing the empty vector. Parasite burdens were determined in the liver and spleen after 4, 6, 8 and 10 weeks following infection via the tail vein. Replicate experiments are shown for this analysis to confirm reproducibility since the kinetics and levels of infection can vary considerably between visceral infection experiments. As shown in Figure 3A, expression of the *L. donovani* Li1040 ortholog in *L. major* gave rise to increased parasite numbers in the liver and spleen, which was most prominent at 8 weeks in the liver, and 6 to 10 weeks in the spleen and this was consistent in the duplicate experiments (Figure 3B, C). We also repeated this analysis by over-expressing the Li1040 gene with the A2 epitope tag removed, to rule out the possibility that the 10 amino acid A2 sequence may have been responsible for the increased virulence associated with the expression of the A2 epitope-tagged Li1040. The Li1040 ortholog gene containing no A2-tag was cloned into *Leishmania* expression vector pLPneo and introduced into *L. major*. As shown in Figure 3C, *L. major* transfected with Li1040 gene with no A2-tag displayed similar visceral infection kinetics in BALB/c mice as *L. major* expressing Li1040 containing the A2-tag. This demonstrated that the A2-tag was not responsible for the increased virulence associated with over-expression of the Li1040 protein in *L. major*.

### The *L. donovani* Li1040 ortholog gene effect on cutaneous infection and proliferation in culture

**Figure 4:**
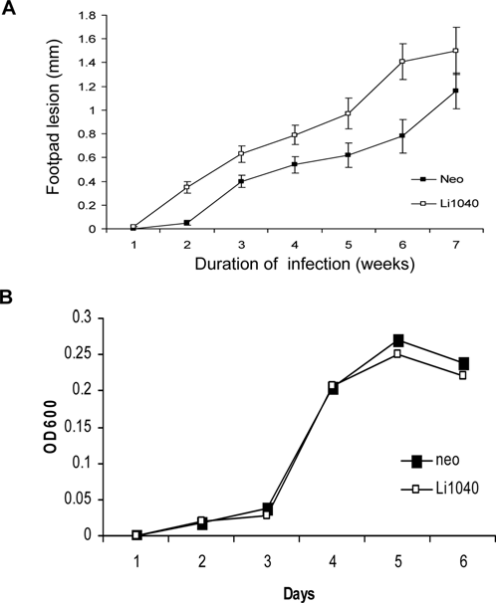
The *L. donovani* Li1040 ortholog gene effect on cutaneous infection and proliferation in culture. A. Footpad infection levels in BALB/c mice with *L. major* expressing the *L. donovani* Li1040 ortholog gene and control *L. major* (neo). B. *In vitro* growth curves of *L. major* promastigotes expressing the *L. donovani* Li1040 ortholog gene or the control vector (neo).

Since *L. major* typically causes cutaneous infections, it was necessary to determine whether expression of the *L. donovani* Li1040 ortholog in *L. major* also increased virulence at cutaneous sites or increased proliferation in cultured promastigotes. BALB/c mice were infected subcutaneously in the rear left footpad with *L. major* expressing the Li1040 ortholog and the control vector containing *L. major* and lesion development were measured weekly. As shown in Figure 4A, expression of the *L. donovani* Li1040 ortholog gene in *L. major* did increase the level of cutaneous infection in the footpad although not to the same extent as was observed in the liver and spleen. Although the *L. donovani* Li1040 ortholog gene enhanced *L. major* virulence *in vivo*, it did not provide a growth advantage to promastigotes in culture (Figure 4B).

### Comparison of the *L. infantum* Li1040 ortholog gene in *L. donovani*, *L. major* and *L. braziliensis*

Our original assumption was that *L. infantum/L. donovani* specific genes would be the most likely to increase visceral infection when expressed in *L. major*, similar to what we observed previously with the *L. donovani* specific A2 gene [12]. It was therefore unexpected that the *L. donovani* Li1040 ortholog gene, which was also present in *L. major* and *L. braziliensis*, was selected for in the visceral organs of BALB/c mice and increased virulence in the liver, spleen and to a lesser extent in the skin.

**Figure 5:**
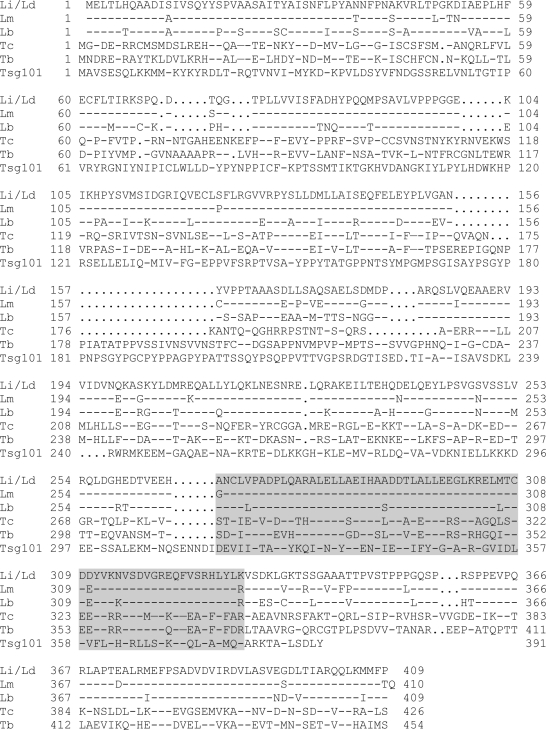
Amino acid sequence alignments of the *L. infantum* Li1040 protein with its homologue proteins. The amino acid sequence of *L. infantum* (Li) (LinJ32_V3.1040) Li1040 protein was aligned with homologue proteins from *L. donovani*, (Ld); *L. major* (Lm) (LmjF32.0985): *L. braziliensis* (Lb), (LbrM32.1080); *T. cruzi* (Tc) (TC00.1047053511725.280); *T. brucei* (Tb) (Tb11.01.5840) and mouse Tsg101 protein (Tsg101). The amino acid sequences of *L. infantum* and *L. donovani* Li1040 proteins are identical. The Vps23_core domains are highlighted.

The ectopic expression of the *L. donovani* Li1040 ortholog in *L. major* may have increased virulence due to increased levels of expression or to sequence variations resulting in a protein with enhanced function. Sequencing of the *L. donovani* Li1040 ortholog gene revealed that it differed by only 5 nucleotides from the *L. infantum* Li1040 gene and none of these nucleotide changes altered the amino acid sequence (Figure 5). Alignment of the *L. infantum/L. donovani* Li1040 protein with the *L. major* and *L. braziliensis* orthologs revealed that Li1040 has 92% identity with the *L. major* ortholog and 82% identity with the *L. braziliensis* ortholog (Figure 5). This is exactly the same percentage (92%) as the genome average amino acid identity between *L. major* and *L. infantum* [5] arguing that there was no selective evolutionary pressure to alter the Li1040 sequence relative to the rest of the genome. The Li1040 ortholog gene is also present in other Kinetoplastids including *Trypanosoma cruzi* (33% identity) and *Trypanosoma brucei* (32% identity) (Figure 5). Interestingly, a Vps23 core domain of the yeast vacuolar protein-sorting protein 23 (Vps23, or Tumor susceptibility gene 101(Tsg101) in human) was identified between amino acid 267 and 331 of the Li1040 protein (see Figure 5 highlighted sequences). The Vps23/Tsg101 proteins have been shown to be involved in protein sorting from endosomes to lysosomes [19],[20], (see more in discussion).

### Endogenous Li1040 ortholog gene expression in *L. donovani* and *L. major*

**Figure 6:**
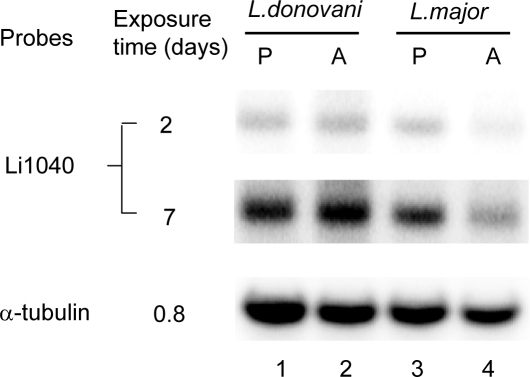
Gene expression analysis of the endogenous Li1040 gene ortholog in wild-type *L. donovani* and *L. major*. Total RNA was extracted from *L. donovani* and *L. major* and subjected to Northern blot analysis with the *L. donovani* Li1040 gene ortholog or the α-tubulin gene as the probes. Lane 1: *L. donovani* promastigotes (P); lane 2: *L. donovani* shifted to amastigote (A) culture conditions for 6 hours; lane 3: *L. major* promastigotes (P); lane 4: *L. major* shifted to amastigote (A) culture for 6 hours. The X-ray film exposure times (0.8, 2 or 7 days) are indicated.

Considering the above observations, it was necessary to investigate the possibility that the endogenous Li1040 ortholog gene was expressed at higher levels in *L donovani* than in *L. major*. Total RNA was extracted from wildtype *L. donovani* and *L. major* cultured under promastigote conditions (27°C, pH 7.4) and amastigotes conditions (37°C, pH 5.5) and subjected to Northern blot analysis with the *L. donovani* Li1040 ortholog gene. As shown in Figure 6, the Li1040 ortholog gene was constitutively expressed in *L. donovani* promastigotes and amastigote-like cultures. The expression level of the Li1040 ortholog gene in *L. major* promastigotes was similar to that in *L. donovani* promastigotes. The lower level of expression in the *L. major* amastigotes culture conditions was likely due to reduced viability of some of the *L. major* cells when cultured briefly at 37°C pH5.5 since the level of the control α-tubulin mRNA was also reduced under these conditions. Compared with the α-tubulin gene, the expression level of Li1040 ortholog gene in both *L. donovani* and *L. major* is low, since only a weak signal was apparent after 2 days of film exposure on the blot and a strong signal was only apparent after 7 days of film exposure. In comparison, a strong signal was apparent for α-tubulin mRNA after less than a day of film exposure with a probe containing the same level of specific radioactivity as the Li1040 ortholog gene probe. Although the level of mRNA is similar, the possibility remains that the Li1040 protein is present in higher levels in *L. donovani* than in *L. major* since the mRNA levels in *Leishmania* generally correlate poorly with the corresponding protein level [21]. Future studies are needed to generate antibodies to the endogenous Li1040 gene product to directly compare protein levels in *L. major* and *L. donovani*.

### Cellular localization of the Li1040 protein in *L. donovani* and *L. major*

**Figure 7:**
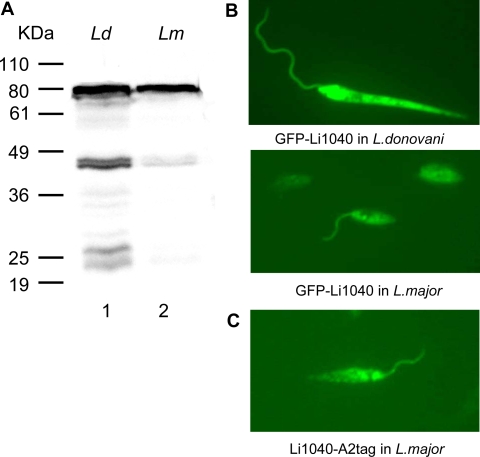
The cellular localization of *L. donovani* Li1040 ortholog protein in *L. donovani* and *L. major*. A. Western blot showing expression of the GFP-Li1040 fusion protein in transfected *L. donovani* and *L. major* promastigotes. B. Cellular localization of GFP-Li1040 fusion protein in *L. donovani* (Top) and *L. major* (Middle) promastigotes determined by GFP fluorescence. C. Cellular localization of the expressed Li1040-A2tag protein in transfected *L. major* promastigotes determined by indirect immunofluoresence with the anti-A2 monoclonal antibody.

Since the sequence analysis of the Li1040 ortholog gene in *L. donovani* and *L. major* showed they are very similar, we sought to determine its cellular localization in these parasites. The *L. donovani* Li1040 ortholog gene was fused with the GFP gene and introduced into *L. donovani* and *L. major*. As shown in Figure 7A, the GFP-Li1040 fusion proteins were expressed at the expected size (791kDa) in *L. donovani* and *L. major*. Fluorescence microscopy revealed that although the GFP-Li1040 ortholog fusion proteins were distributed throughout the cell including the flagella in both *L. major* and *L. donovani* transfectants, the majority of GFP-Li1040 fusion protein appear to be present as cytoplasmic aggregates (Figure 7B). Similar localization of the A2-tagged *L. donovani* Li1040 ortholog protein was shown in transgenic *L. major* using anti-A2 Mabs (Figure 7C). Interestingly, comparable cellular appearance and distribution were reported for the mammalian Tsg101 protein, the potential homolog of *Leishmania* Li1040 [22].

### Over expression of the *L. major* Li1040 ortholog gene (Lm0985) in *L. major*

**Figure 8:**
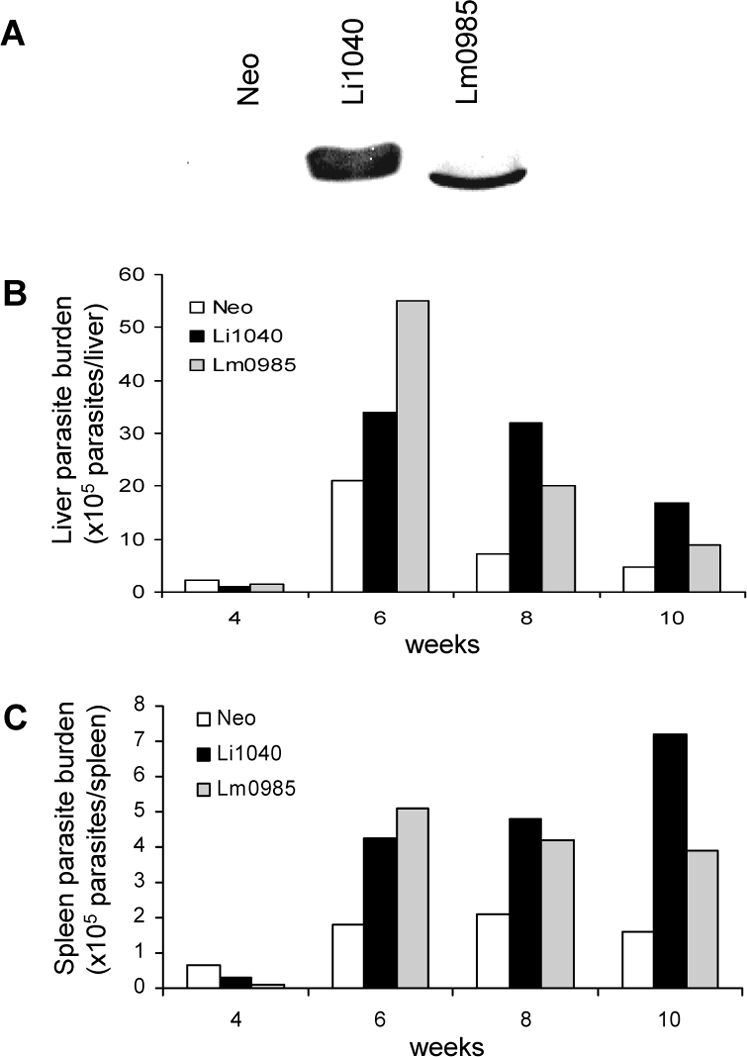
Over expression of *L.major* Li1040 ortholog gene (Lm0985) in *L. major*. A. Western blot showing the expression levels of Li1040 and Lm0985 proteins (both with A2 tag) in transfected *L. major* promastigotes. The liver (B) and spleen (C) parasite burdens of mice infected with *L. major* over expressing Li1040 or Lm0985, and *L. major* containing control vector (Neo) were determined after 4, 6, 8 and 10 weeks after infection.

The above described experimental analyses suggest that the increased expression level of plasmid derived Li1040 may have played a greater role in increasing parasite virulence than the sequence differences between the corresponding *L. donovani* and *L. major* ortholog genes. We attempted to address this issue by over-expressing the *L. major* Lm0985 gene in *L. major*. Lm0985 is the *L. major* ortholog of *L. infantum/donovani* Li1040. As shown in Figure 8A, epitope tagged *L. major* Lm0985 was detectable in transfected *L. major*. Over-expression of the *L. major* Lm0985 and *L. donovani* Li1040 othologs in transgenic *L. major* both resulted in increased parasite levels in the liver and spleen at various times following infection (Figure 8B, C). The generally high levels of infection seen with the Li1040 transgenic *L. major* parasites relative to the Lm0985 transgenic parasites could have however been due to the higher expression levels of Li1040 relative to Lm0985 (Figure 8A). Taken together these results argue that over-expression of either Lm0985 or Li1040 results in increased parasite virulence. It was also noteworthy that, although there was approximately a 4 fold increase in spleen parasite numbers with the Li1040 gene expressing *L. major* compared to the control Neo *L. major* parasites at 10 weeks following infection (Figure 8C), this difference was less than the previous 3 independent infection experiments shown in Figure 3. This highlights the importance of carrying out multiple repeat independent infection experiments which taken together confirm that over-expression of Li1040 results in increased virulence.

## Discussion

In the present study we have developed an experimental approach to generate and follow transgenic *L. major* expressing *L. donovani* genes *in vivo* in BALB/c mice. Rational for this study comes from our previous observations where expression of the *L. donovani* specific A2 gene increased *L. major* survival in visceral organs [12] and we therefore anticipated that additional *L. donovani/L. infantum* specific genes could also increase *L. major* virulence in the visceral organs. *L. major* expressing the *L. donovani* Li1040 ortholog gene was selected for in the spleen of BALB/c mice and further shown to dramatically increase *L. major* parasite numbers in the liver and spleen and to a much lesser extent in the skin. This outcome was somewhat unexpected since the Li1040 ortholog gene was subsequently established to be present in *L. major* and *L. braziliensis* in addition to *L. donovani* and *L. infantum*. This revealed that genes ubiquitously present in different *Leishmania* species could also have a dramatic effect on parasite tropism and virulence.

It is noteworthy that Li1040 expressing transgenic *L. major* were rapidly selected for *in vitro* after 3 days when placed under amastigote culture conditions at 37°C and pH 5.5. Over-expression of the Li1040 ortholog however did not enhance promastigotes proliferation in culture at 27°C and pH 7.4 (Figure 4B). This suggests that the Li1040 product enables amastigotes to survive under conditions associated with host macrophage phagolysosomes. This was consistent with the observation that Li1040 ortholog expressing *L. major* were likewise selected for *in vivo* after several weeks in the liver and spleen of BALB/c mice (Figure 2). Although these observations suggest that Li1040 plays a greater central role in the amastigote stage, it also appears to be essential for survival as promastigotes since repeated attempts to generate homozygous *L. donovani* Li1040 gene deletions in promastigotes have so far been unsuccessful.

It was interesting to find that the Li1040 protein contains the Vps23 core domain of the Vps23 and Tsg101 proteins in yeast and human respectively. Vps23 is one of the four protein subunits (Vps23, Vps28, Vps37 and Mvb12) of the yeast ESCRT-I (Endosomal Sorting Complex Required for Transport) complex, which forms the complex driving protein transport from endosomes to lysosomes [19],[20]. The core domain of the Vps23 has been shown to be essential for formation of ESCRT-I complex [19],[20]. Moreover, the homologs of the three other subunits of ESCRT-I complex (Vps28, Vps37 and Mvb12) also appear to be present in the *Leishmania* genome (LinJ36_V3.5400 encodes a homolog of Vps28, and several potential homologue proteins for Vps37 and Mvb12 are also present in *Leishmania*, data not shown). Since all 4 ESCRT-1 subunits appear to be present in *Leishmania*, this suggests that, similar to Vps23, Li1040 may also be involved in protein transport. In higher eukaryotes, the Tsg101 (tumour susceptibility gene 101) plays an essential role in embryonic development [23],[24] and loss of Tsg101 in cell lines is associated with neoplastic transformation [25] confirming it plays an essential role in cell biology.

It is noteworthy that the focus of this study was on genes whose expression was selected for in the parasites isolated from the spleen of BALB/c mice. Although this approach proved successful to identify the ability of the Li1040 gene to increase virulence, this does not rule out the possibility that some of the other species-specific genes can also increase virulence. Since there are relatively few species-specific genes, it may be possible to further study the role of individual genes without performing the *in vivo* selection used in this study. For example, the cyclopropane fatty acyl phospholipid synthase (CFAS) gene is present in *L. infantum* and *L. braziliensis* but not *L. major*. The CFAS gene in *Mycobacterium tuberculosis* (Mtb) has been shown to modify cell surface glycolipids, which promotes an inflammatory response and granuloma formation (Table 1) [26]. It would be interesting to determine whether CFAS affects *Leishmania* surface glycolipid composition and virulence even though it was not enriched for following *in vivo* selection in mice in this study. The gene encoding Sec14 cytosolic factor is present in *L. infantum* but is a pseudogene in *L. braziliensis* and absent in *L. major* (Table 1) [7]. The Sec14 cytosolic factor has been implicated in the release of secretory vesicles from the trans-golgi network [27] and therefore may influence cell-surface molecule expression in *L. infantum* and affect host-parasite interactions. These examples and others could be tested individually by transfecting the *L. infantum* genes into *L. major* and studying the phenotype of the resulting transgenic parasites. We are currently focusing on additional *L. infantum* specific genes, which are absent in both *L. major* and *L. braziliensis* and introducing these into *L. major* to assay for changes in virulence as described in this study. The genetic determining factor(s) controlling tropism and virulence could however be widely embedded throughout these different genomes involving a combination of species-specific genes, posttranscriptional regulation and gene polymorphisms. The Li1040 gene identified in this study may be among those playing a major role in virulence and tropism.
